# Proteomic Data Integration Highlights Central Actors Involved in Einkorn (*Triticum monococcum* ssp. *monococcum*) Grain Filling in Relation to Grain Storage Protein Composition

**DOI:** 10.3389/fpls.2019.00832

**Published:** 2019-07-04

**Authors:** Emmanuelle Bancel, Titouan Bonnot, Marlène Davanture, David Alvarez, Michel Zivy, Pierre Martre, Sébastien Déjean, Catherine Ravel

**Affiliations:** ^1^UMR GDEC, Institut National de la Recherche Agronomique (INRA), Université Clermont Auvergne, Clermont-Ferrand, France; ^2^UMR1095, Genetics Diversity and Ecophysiology of Cereals, Clermont Auvergne University, Clermont-Ferrand, France; ^3^UMR GQE, Institut National de la Recherche Agronomique (INRA), Centre National de la Recherche Scientifique (CNRS), Agro ParisTech, Université Paris-Sud – Université Paris-Saclay, Gif-sur-Yvette, France; ^4^Institut de Mathématiques de Toulouse, UMR5219 Université de Toulouse, Centre National de la Recherche Scientifique (CNRS), Toulouse, France

**Keywords:** albumin-globulin, data integration, nitrogen, proteomic, grain development, storage protein, sulfur, *Triticum monococcum*

## Abstract

Albumins and globulins (AGs) of wheat endosperm represent about 20% of total grain proteins. Some of these physiologically active proteins can influence the synthesis of storage proteins (SPs) (gliadins and glutenins) and consequently, rheological properties of wheat flour and processing. To identify such AGs, data, (published by Bonnot et al., 2017) concerning abundance in 352 AGs and in the different seed SPs during grain filling and in response to different nitrogen (N) and sulfur (S) supply, were integrated with mixOmics R package. Relationships between AGs and SPs were first unraveled using the unsupervised method sparse Partial Least Square, also known as Projection to Latent Structure (sPLS). Then, data were integrated using a supervised approach taking into account the nutrition and the grain developmental stage. We used the block.splda procedure also referred to as DIABLO (Data Integration Analysis for Biomarker discovery using Latent variable approaches for Omics studies). These approaches led to the identification of discriminant and highly correlated features from the two datasets (AGs and SPs) which are not necessarily differentially expressed during seed development or in response to N or S supply. Eighteen AGs were correlated with the quantity of SPs per grain. A statistical validation of these proteins by genetic association analysis confirmed that 5 out of this AG set were robust candidate proteins able to modulate the seed SP synthesis. In conclusion, this latter result confirmed that the integrative strategy is an adequate way to reduce the number of potentially relevant AGs for further functional validation.

## Introduction

The total proteins in cereal grains mainly consist of grain storage proteins (SPs) and metabolic proteins. In wheat, SPs gliadins and glutenins, make up 60–80% of the total protein content. Glutenins are classified as high-molecular-weight or low-molecular-weight glutenin subunits (HMW-GS and LMW-GS, respectively) and gliadins are divided into α-, γ-, and ω-gliadins. Grain SPs have been extensively studied in contrast to metabolic proteins, which have not yet been well characterized. Metabolic proteins correspond to the soluble albumin-globulin (AG) fraction, which contributes to 15–20% of the total grain protein content in bread wheat (Shewry and Halford, 2002). The effect of developmental changes in the metabolic proteins of wheat endosperm was first investigated using two- dimensional gel electrophoresis, image analysis and mass spectrometry (2-DE/MS/proteomics; Vensel et al., 2005; Merlino et al., 2009; Debiton et al., 2011; Tasleem-Tahir et al., 2012). Recently, several quality-related albumins and globulins were identified in an elite Chinese wheat cultivar (Dong et al., 2012) and changes occurring in durum wheat with a focus on allergens were analyzed (Arena et al., 2017). These authors based their methodology on two-dimensional electrophoresis (2-DE) and two-dimensional differential gel electrophoresis (2D-DIGE). However, these two approaches have several limitations such as identification of a low number of proteins. Other methods, such as isobaric tags for relative and absolute quantitation (iTRAQ)-based quantitative proteome approach (Ma et al., 2014) have also been used to analyze the proteome of wheat grain development. Recently, Victorio et al. (2018) used nanoUPLC and Ultra Definition Mass Spectrometry to identify and quantify soluble proteins in nine bread wheat cultivars of low, medium or high quality.

The nutritional status of the plant is known to significantly affect protein synthesis and accumulation in wheat. In particular, nitrogen (N) and sulfur (S) nutrition have been reported to affect several enzymes in wheat grain. For example, a glyceraldehyde-3-phosphate dehydrogenase and a serpin were both increased using a treatment combining high N and low S (Flæte et al., 2005). Modifications of AG synthesis and accumulation in the grain due to different levels of N and S likely disturbed the balance of cell functions that differentially affected carbohydrate and amino acid metabolism, and transport (Bonnot et al., 2017).

N and S supplies together influence the N to S ratio in the grain, leading to significant changes in grain SP composition at maturity (Dai et al., 2015; Bonnot et al., 2017). N supply induces changes in the rate and duration of SP accumulation during grain filling. SP concentration is increased by higher N supply and the proportion of different SP classes is modified in response to N application (Chope et al., 2014; Bonnot et al., 2017; Yu et al., 2017). S deficiency also has strong impacts on SP composition, which is associated with alterations of dough rheology (Zhao et al., 1999). S fertilization improved all major parameters of baking quality in wheat by increasing the content of S-containing amino acids (Järvan et al., 2008). S deficiency promotes the synthesis and accumulation of S-poor proteins such as ω-gliadin and HMW-GS at the expense of S-rich proteins (Zhao et al., 1999; Flæte et al., 2005; Bonnot et al., 2017).

In contrast to this, the effects of S on the synthesis and accumulation of HMW and LMW glutenins are opposite. Therefore, S deficiency decreases the total amount of polymeric proteins because LMW are the major components of glutenins. Changes were brought on by an imbalance of S-modified protein composition which is associated with alterations of dough rheology (Zhao et al., 1999). S fertilization improved all major parameters of baking quality in wheat by increasing the content of S-containing amino acids (Järvan et al., 2008).

In summary, N and S supplies together influence the N to S ratio in the grain leading to significant changes in SP composition at maturity (Bonnot et al., 2017).

In order to gain insights into the contribution of AGs to grain storage composition, we analyzed the large-scale proteome dataset for Einkorn (*Triticum monococcum* ssp. *monococcum*) described in Bonnot et al. (2017). Einkorn is an ancestral wheat whose diploid genome is a sister to bread wheat (*T. aestivum*) genome A (Marcussen et al., 2014) and for which genomic resources are available, making it a suitable species in which to explore proteome responses to N and S nutrition. We used the integrative methods implemented in the R package MixOmics (Lê Cao et al., 2016) and put forward by Duruflé et al. (2018) that take into account all quantified proteins and not only those differentially expressed as in classical omics analyses. We identified 18 important AGs. We provided a statistical validation of five of them in bread wheat by association analysis and seven of these identified AGs have already been identified as potential candidates in a network analysis based on association rule discovery (Bonnot et al., 2017). This cross-validation confirms the ability of these integrative methods to identify robust candidate entities, which could be considered for functional analysis.

## Materials and Methods

### Plant Material and Grain Sampling and Processing

The einkorn wheat (*T. monococcum*) accession ERGE 35821 was cultivated in a greenhouse as described previously (Bonnot et al., 2017). Briefly, plants were grown in PVC columns filled with a 2:1 (v:v) mixture of washed perlite and river sand and arranged to form an homogenous canopy with a density of 512 plants m^-2^. The experimental design was a randomized complete block design with four blocks and four treatments. Until anthesis, each PVC column received 167 mL days^-1^ of a modified Hoagland’s nutrient solution (Castle and Randall, 1987) containing 3 mM N and 0.1 mM S prepared with demineralized water. At anthesis, when N and S demand is low, the nutrient solution was replaced with demineralized water to avoid excess build-up of N and S compounds in plants or potting substrate. Then, from 200 to 700°C days after anthesis, four N and S treatments were applied: N0S0, nutrient solution with no N or S; N, 6 mM N with no S; S, low N (0.5 mM) and high S (2 mM); or NS, high N (6 mM) and high S (2 mM) as described by Bonnot et al. (2017) (Supplementary Figure 1).

Main-stem ears were tagged at the date of anthesis and only grains from the middle of the tagged ears were harvested every 100°C days (approximately every 5 days) from 100 to 1000°C days after anthesis. Thermal time was calculated as cumulative degree-days above 0°C. For each treatment and sampling date, grains from four to 10 main-stem ears were collected per replicate, depending on the grain developmental stage. Except for those used to determine grain dry mass, samples were frozen immediately in liquid N_2_, and then stored at -80°C until use. Four biological replicates of whole grains were used to determine grain dry mass and SP composition, and three to identify and quantify AG proteins.

### Measurement of Grain Traits

Gliadin and glutenin proteins were sequentially extracted from 100 mg of wholemeal flour milled from each replicate of grain sampled between 300 and 1000°C days after anthesis, as described by Plessis et al. (2013), Bonnot et al. (2017). Gliadin classes and glutenin subunits were separated and quantified by reverse phase high-performance liquid chromatography following the procedure described by Dai et al. (2015). Chromatograms were processed with ChemStation 10.1 software (Agilent Technologies) and the peaks corresponding to each of the four gliadin classes and the two glutenin subunits were identified following the observations of Wieser et al. (1998).

### Analysis of Metabolic Proteins

Metabolic proteins were extracted from whole grains as described in Bonnot et al. (2017). Briefly, proteins were extracted for 2 h at 4°C in 10 mM sodium phosphate, 10 mM NaCl, pH 7.8, supplemented with a cocktail of plant protease inhibitors (Sigma, St. Louis, MO, United States). After centrifugation at 8,000 × *g* for 20 min at 4°C, proteins in the supernatant were precipitated with ice-cold acetone for 2 h at -20°C. After centrifugation at 10,000 × *g* for 5 min at 4°C, the resulting pellets were washed three times in ice-cold acetone then dried at room temperature and afterward stored at -20°C. The precipitated AG proteins were suspended in a solubilization buffer (0.1% ZALS I, 6 M urea, 2 M thiourea, 10 mM DTT, 30 mM Tris–Hcl pH 8.8, 50 mM NH_4_HCO_3_). Proteins were digested in-solution by trypsin and resulting peptides were analyzed by LC-MS/MS using a nanoLC Ultra system (Eksigent) and a Q-Exactive mass spectrometer (Thermo Electron).

### Protein Identification and Quantification

The mass spectrometry proteomic data were first published in Bonnot et al. (2017) and have been deposited to the ProteomeXchange Consortium (Deutsch et al., 2017) via the PRIDE (Vizcano et al., 2016) partner repository with the dataset identifier PXD006058.

Proteins were identified by matching peptides using the Uniprot protein database version 2014_07 limited to the *Triticum* genus using X!Tandem ^1^ and X!TandemPipeline (Langella et al., 2017) as described in Bonnot et al. (2017).

Functional classification was established on the basis of gene ontology (GO) information rules provided by Uniprot (Ashburner et al., 2000). Proteins that had no functional annotation or GO information were analyzed with Blast2GO (version 3.2, Conesa et al., 2005) in order to assign potential function.

Peptide quantification was performed by integration of extracted ion current (XIC) using MassChroQ (Valot et al., 2011). Protein relative quantities were computed by summing the XIC value of specific peptides, i.e., peptides that were not shared by different proteins. Only proteins quantified with at least two peptides were selected for subsequent analysis.

### Data Integration and Discriminant Analysis

**FIGURE 1:**
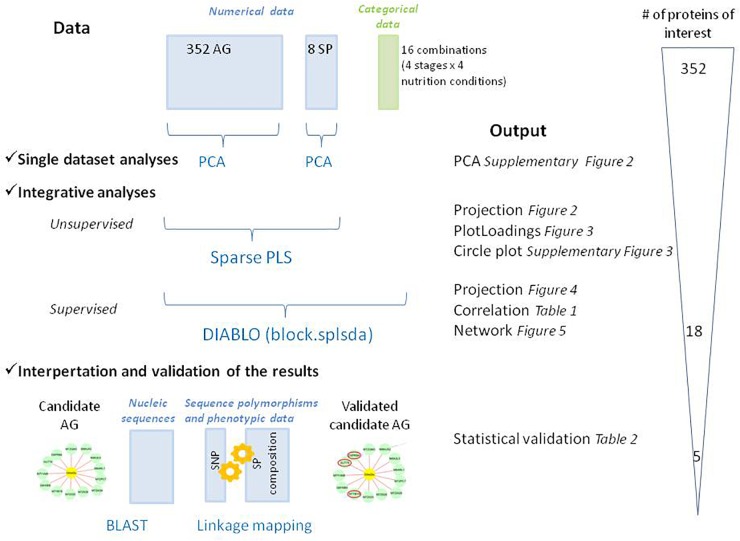
Schematic overview of the methods used to co-analyze the different datasets in this study. The graphical outputs detailed in the results section are indicated with their reference in the text. PCA, Principal Component Analysis; PLS, Partial Least Squares regression; DIABLO: Data Integration Analysis for Biomarker discovery using Latent variable approaches for “Omics studies,” DA, Discriminant Analysis; BLAST, Basic Local Alignment Search Tool. Qualitative (categorical data) and quantitative (numerical data) blocks are represented in green and blue, respectively. The number of proteins of interest at each step was indicated.

Omics data integration was performed using the R package MixOmics (version 6.3.2), which offers a whole range of multivariate methods to explore and integrate biological datasets (Lê Cao et al., 2009; Rohart et al., 2017). Our datasets consisted of two sets (the AG and the SP sets) measured on the same samples with three independent biological replicates. The AG dataset included 352 proteins present in all samples and in the 2018_12 version of Uniprot protein database. The SP dataset included the quantity per grain of total glutenin subunits and gliadins, the total quantity per grain for each glutenin and gliadin classes i.e., HMW and LMW, α-, γ-, and ω-gliadins. These two datasets (values were scaled) were explored according to the workflow presented (Figure 1). We first investigated independently each dataset by Principal components analysis (PCA). Then, both datasets were analyzed conjointly with an unsupervised method, the sparse Partial Least Squares (sPLS) regression (Lê Cao et al., 2008). The PLS algorithm searches for the largest covariance between orthogonal components, which are linear combinations of the variables from both datasets studied. Sparsing leads to the selection of variables, which are the most involved in the relationships between the biological datasets. We kept 15 variables for each dataset (i.e., all the variables for the SP set). The *plotIndiv()* function was used to project the samples onto the PCA and sPLS components. For sPLS, two plots can be obtained because a set of PLS-components is calculated on each dataset. Mathematically, it corresponds to two subspaces spanned by the variables in each dataset. A mean subspace can result from them. In this third subspace, sample coordinates were averaged from the subspaces spanned by each dataset. The *plotloading()* function was used to visualize the loading vector of the sPLS analysis and represent the contribution of the variables kept for each component. Then, we used the *block.splda()* function from DIABLO (Data Integration Analysis for Biomarker discovery using Latent variable approaches for Omics studies) (Singh et al., 2019) to identify correlated (or co-expressed) variables measured on our two datasets, which also explain the categorical variable of interest used to supervise the analysis. This latter analysis was supervised by crossing the developmental stage of the grain (four stages) and the nutrition (four nutritions) resulting in 16 combinations. Networks were built using the function *network()* from the package mixOmics to show the correlations (*r* > |0.8|) between selected features. Cytoscape software version 3. 7. 1 (Shannon et al., 2003) was used to visualize the network.

### Statistical Validation

A statistical validation of AGs involved in the network was undertaken by linkage mapping. The coding sequence of each AG in the network was retrieved from Uniprot and then blasted against the wheat pseudomolecule (IWGSC RefSeq v1.1 database^2^). The BLAST results were analyzed to find the coordinates of the bread wheat homologs on the pseudomolecule. Taking into account flanking sequences could be relevant because of linkage disequilibrium range in wheat and their role in gene expression. Therefore, gene regions were increased by 1,500 nucleotides upstream and downstream. Single Nucleotide polymorphisms (SNPs) located in these increased regions were extracted from the set developed by Rimbert et al. (2018). Their genotyping data were retrieved for the 196 lines from the agronomic part of the INRA worldwide core collection (Bordes et al., 2011), which was phenotyped for grain SP composition (Plessis et al., 2013). Association study was performed with the mixed model (Yu et al., 2006) implemented in the R package GWAS from rrBLUP (Endelman, 2011) comprising the kinship matrix (K) that accounts for relatedness among accessions to limit spurious associations. The Leave One Chromosome Out (LOCO) approach was used to construct different kinship matrices by testing each chromosome and leaving out the SNPs on the chromosome being tested (Yang et al., 2014). Each off-target variants (OTV) was recoded to create two biallelic markers (a first one with the two nucleotide variants and a second one coded as presence for the nucleotide variants) or absence (for no call). Markers with a minor allele frequency < 0.05, and a missing rate > 0.1 were discarded. The significance of associations was tested with an *F*-test. Associations were judged significant at *P* < 0.001.

## Results

The AG proteome of the grain in development in response to N and S nutrition was first analyzed by Bonnot et al. (2017) to emphasize components involved in SP synthesis. Here, both dataset (AGs included 352 quantified proteins present in all samples and still present in Uniprot database, and SPs) were integrated to find candidate AGs related to seed storage synthesis.

### Data Structuration

**FIGURE 2:**
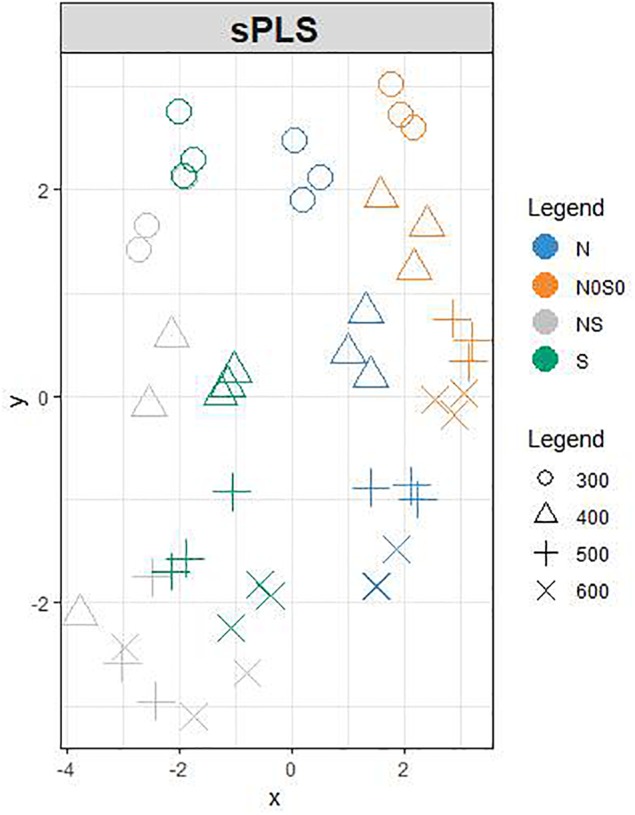
Projection of each sample based on the sPLS analysis onto the subspace in which the coordinates are averaged from AG and SP subspace. AGs and SPs are characterized from grain at 300 (o), 400 (△), 500 (+), and 600 (x) °C days as indicated by the numbers. They received after anthesis a nutrient solution with no N and no S (N0S0, orange); N with no S (N, blue); S with low N (S, green) or high S and high S (NS, gray). The colors of the individuals have been added after the analysis.

**FIGURE 3:**
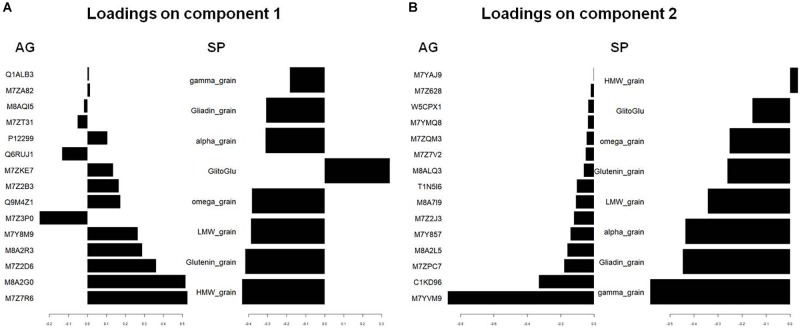
Visualization by plotLoadings after a supervised classification by sPLS of the variables that are the best discriminating on component 1 **(A)** and component 2 **(B)**. The figure shows coefficient weight of the features selected on component 1 and component 2, with the maximal variable weight (numerical scale) on each feature in the two datasets blocks: AG and SP. The Uniprot identification is used for AGs. HMW_, LMW_, glutenin_grain indicate the quantity per grain of LMW-, HMW-, and total glutenins. Alpha_, gamma_, omega_, and gliadine_grain indicate the quantity per grain of Alpha_, gamma_, omega_, and total-gliadins. GlitoGlu is the gliadin to glutenin ratio.

We used PCA (Supplementary Figure 2) and sPLS (Figure 2) to observe the structuration of our datasets. Individuals were projected in the subspace consisting of the AG and SP sets and then in a subspace in which coordinates are averaged from the two precedent subspaces (Figure 2). Roughly, grain developmental stages were discriminated in the AG subspace with the first two principal components explaining up to 54% of the total variance, while nutrition conditions were better discriminated in the subspace defined by SPs with 95% of the variability explained by the two first principal components (Supplementary Figure 2). The developmental stages and nutrition conditions are rather well discriminated in the mean subspace resulting from sPLS, which integrated both AG and SP datasets (Figure 2). In this subspace, the *x*-axis discriminated the nutrition treatments. The two opposite nutrition conditions (N0S0 and NS) were at both extremities of this axis, which could be considered as an S-axis. Indeed, nutrition conditions with S (NS and S) have negative *x*-coordinates unlike conditions lacking S (N and N0S0). The *y*-axis discriminated the developmental stages with 300 and 600°C days having, respectively, positive and negative coordinates. The loading weights indicated that SPs strongly contributed to the first two components (Figure 3). For the first component, all SP variables had an absolute weight >0.2, a positive weight being observed only for the gliadin to glutenin ratio. The quantity of HMW glutenin was the only SP variable with a positive contribution for the second component. This contribution was light. Six AGs (out of the 15 kept for this component) have a contribution for the first component with an absolute weight >0.2. The strongest weights (>0.5) are found for M7Z7R6 and M8A2G0. M7Z3P0 was the AG with the most important negative contribution for this axis. M7YVM9 had the strongest negative contribution to the second axis (>-0.8). According to these contributions for the two first components, the *x*-axis of the circle plot (Supplementary Figure 3) is mostly explained by all variables related to SP composition, which appear to be influenced by the nutritional status. The gliadin to glutenin ratio was negatively correlated with the other SP variables. We observed that some AG variables are close in the correlation circle plot to SP variables (Supplementary Figure 3). For instance, the gliadin to glutenin ratio is close to M7ZKE7 (Uncharacterized protein/glutathione transferase domain) and M8A2R3 (Uncharacterized protein/Ricin B-like lectin EULS3-like family).

**FIGURE 4:**
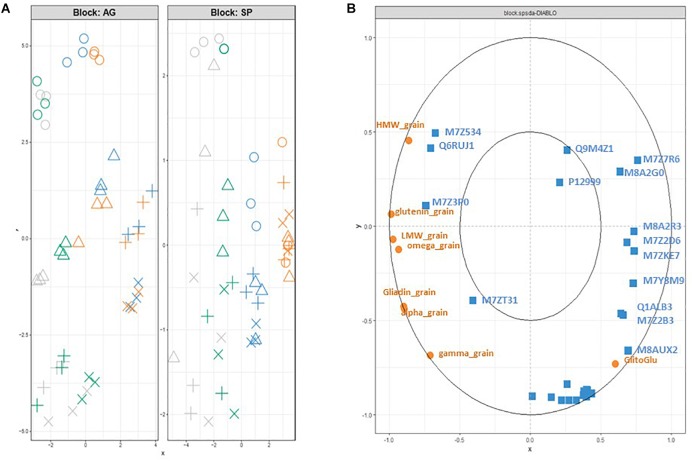
Projection of each sample based on the DIABLO analysis (block.plsda) onto **(A)** the subspaces spanned by the components of each dataset (X and Y blocks for AG and SP dataset, respectively). AGs and SP are characterized from grain at 300 (o), 400 (△), 500 (+), and 600 (x) °C days. They received after anthesis a nutrient solution with no N and no S (N0S0, orange); N with no S (N, blue); S with low N (S, green) or high S and high S (NS, gray). **(B)** Correlation circle plot for the kept variables of the block.splda analysis in DIABLO (15 AGs and all the variables (8) for the SP dataset). The variables are selected on component 1 and 2 from the two datasets (AG in blue, PR in orange). The Uniprot identification is used only for the 15 AGs. HMW_, LMW_, glutenin_grain indicate the quantity per grain of LMW-, HMW- and total glutenins. Alpha_, gamma_, omega_ and gliadin_grain indicate the quantity per grain of alpha_, gamma_, omega_ and total-gliadins. GlitoGlu is the gliadin to glutenin ratio.

As expected, the DIABLO analysis with the *block.splda()* function improved the structuration resulting from the sPLS analysis as it is a supervised method aiming at discriminating the 4 groups (Figure 4). In the subspace of AGs, the *x-* and *y*-axis discriminated the nutrition treatments and the developmental stages, respectively (Figure 4A). The nutrition treatments were clearly discriminated in the SP subspace (Figure 4A), where the separation of the development stage was better for the S conditions (NS and S) than for the N conditions (N0S0 and N). The correlation circle plot (Figure 4B) highlighted the contribution of selected variables to each component. SPs are related to the *x*-axis with a clear opposition between the quantity per grain of glutenin subunits (HMW and LMW) and total glutenin, and the gliadin to glutenin ratio. Some AGs appeared to be highly correlated with specific SP variables. For instance, M7Z534 (Glutaredoxine-C8) and Q6RUJ1 (Glutamine synthetase) are positively correlated with the quantity per grain of HMW glutenins. Similarly, M8AUX2 (Uncharacterized protein/caleosin related domain) is positively correlated with the gliadin to glutenin ratio. Many AGs were projected as a cluster in the correlation circle plot (Figure 4B). The *x*-axis coordinate for this cluster is near 0 indicating that these AGs are not affected by nutrition status. The *y*-coordinate is strongly negative indicating a higher accumulation in the late developmental stages.

### Network Analysis Reveals Albumins-Globulins Involved in Grain Storage Protein Accumulation

**FIGURE 5:**
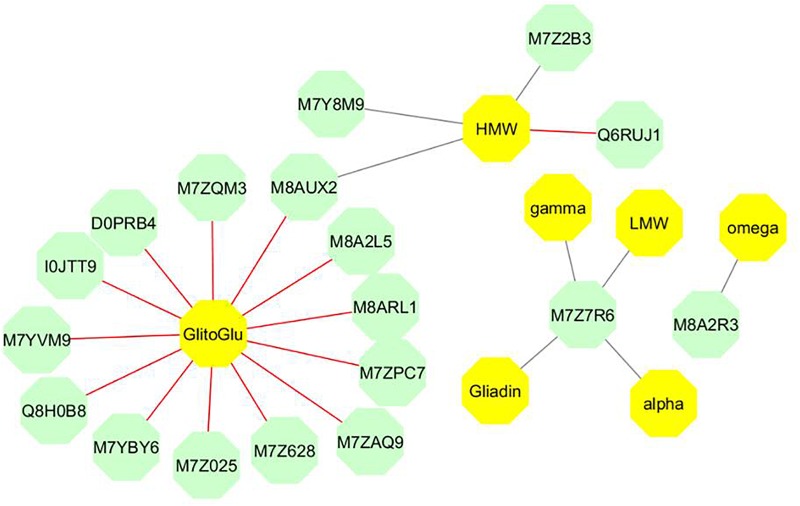
Network produced by the block.plsda in DIABLO. Each color represents a type of variable (green and bright yellow for AGs and SPs, respectively). The correlation cut off applied was 0.8. The optimal components were considered in the network, which is bipartite i.e., only links between two variables of different types are drawn. The color of edges indicates the sign of the correlation: red and gray correspond to positive and negative correlation, respectively (Table 1). The Uniprot identification is used for AGs. HMW and LMW indicate the quantity per grain of HMW- and LMW- glutenins. Alpha, gamma, omega and Gliadin are the quantity per grain of α-, γ-, ω, and total-gliadins, respectively. GlitoGlu is the gliadin to glutenin ratio.

We analyzed the results of the two-block analysis performed with the block.plsda from DIABLO, supervised by the nutrition × stage combinations, where the AG and SP datasets were quantitative variables with three independent biological replicates analyzed. Fifteen AG proteins were selected on each component and connected to variables present in the SP block. On the network (Figure 5 and Table 1), positive and negative correlations > |0.8| between variables from 18 AG to 7 SP variables from datasets were simultaneously represented. Three sub-networks were identified.

**Table 1:** Proteins highlighted in the network.

Uniprot ID	Protein name^a^	LMW_glutenin	HMW_glutenin	α_gliadin	γ_gliadin	ω_gliadin	Gliadin_grain	GlitoGlu
D0PRB4	Peroxiredoxin							0.91
I0JTT9	Glutamate decarboxylase							0.92
M7Y8M9	Translocon-associated protein subunit beta		-0.83					
M7YBY6	Basic endochitinase C							0.83
M7YVM9	Chitinase 1							0.82
M7Z025	Ankyrin repeat domain-containing protein 2							0.85
M7Z2B3	Uncharacterized protein/transmembrane 214-B^∗^		-0.80					
M7Z628	1-Cys peroxiredoxin PER1							0.86
M7Z7R6	Uncharacterized protein/isoflavone reductase^∗^	-0.82		-0.83	-0.82		-0.82	
M7ZAQ9	Oxalate oxidase 2							0.88
M7ZPC7	Heat shock cognate 70 kDa protein							0.90
M7ZQM3	Globulin-1 S allele							0.91
M8A2L5	Uncharacterized protein/water stress induced protein ^∗^							0.91
M8A2R3	Uncharacterized protein/Ricin B-like lectin EULS3-like family^∗^					-0.81		
M8ARL1	Uncharacterized protein/Oil body-associated 1A^∗^							0.90
M8AUX2	Uncharacterized protein/caleosin 2^∗^		-0.91					0.88
Q6RUJ1	Glutamine synthetase		0.85					
Q8H0B8	Cold regulated protein							0.89


*Accessions and protein names given by Uniprot were indicated. Only the positive and negative correlations values (>|0.8|) obtained between AGs and SP with the DIABLO approach were given. Storage proteins expressed by grain (mg N grain-1), GlitoGlu, gliadin to glutenin ratio. ^*a*∗^Indicates function assigned by BLAST for uncharacterized proteins.*

The main sub-network includes 16 AGs, which were connected to two SP variables (gliadin to glutenin ratio and the quantity of HMW glutenins per grain). In this sub-network, the correlations between the gliadin to glutenin ratio and the 13 AGs linked with the SP variables were all positive (Table 1). Out of these 13 metabolic proteins, two were uncharacterized proteins (M8A2L5, M8ARL1), which nevertheless contained annotated domains. M8ARL1 has an oil body associated A1domain and M8A2L5 classified in water stress induced proteins. The 11 remaining AGs have an annotation. M7ZQM3 is a globulin 1S. I0JTT9 is a glutamate decarboxylase known to be the rate-limiting enzyme for γ-aminobutyric acid (GABA) synthesis. M7YBY6 and M7YVM9 are two chitinase proteins related to the pathogenesis-related proteins and also involved in plant abiotic stress responses. M7ZAQ9 is an oxalate oxidase, which catalyzes the oxidation of oxalic acid to CO_2_, Ca ^2+^, and H_2_O_2_, and is stored as insoluble calcium salt upon reaction with molecular oxygen (Lane et al., 1993). Q8H0B8 is a cold regulated protein, member of the phosphatidylethanolamine-binding protein (PEBP) superfamily. M7Z025 is an ankyrin repeat domain-containing protein 2. Ankyrin repeats are present in many proteins of eukaryotes, prokaryotes and some viruses and they function as protein–protein interaction domains. D0PRB4 (Peroxiredoxin) and M7Z628 (1-Cys peroxiredoxin PER1) are known to play a significant role in protection systems such as peroxidases or molecular chaperones; M7ZPC7 is, a heat shock cognate 70 kDa protein, able to act as a chaperone protein, playing major roles in the prevention of target protein aggregation and promotion of their correct folding. The last AG with an annotation was M8AUX2, a caleosin which is, like caleosin-related sequences, a lipid-associated protein found in a wide range of higher plants. M8AUX2 played a particular role in the sub-network as the AG connecting the two SP variables with a positive correlation to the gliadin to glutenin ratio and a negative one to the quantity of HMW glutenins. This latter SP variable was negatively correlated to M7Y8M9, a translocon-associated protein subunit beta and M7Z2B3, a transmembrane 214-B domain, involved in endoplasmic reticulum-stress induced apoptosis, and positively correlated with Q6RUJ1, a glutamine synthetase.

The second sub-network concerned M7Z7R6, an uncharacterized protein, similar to some isoflavone reductase, which showed strongly negative correlations with all S-rich SPs (α-, γ-, total-gliadins, and LMW glutenins). This grouping could result from the common response to S supply of these SP classes.

The last sub-network was the smallest. It included two negatively correlated components, the ω-gliadin (S-poor SPs) and M8A2R3. M8A2R3, an uncharacterized protein, presents a domain related to ricin B-like lectin EULS3-like family. This domain is found in a group of plant proteins called lectins which bind carbohydrates.

We clearly observed that abundance of the 18 AGs from the network was more impacted by the grain developmental stage than by the N and S treatments (Supplementary Figure 4). All of them, except M7Y8M9 (Translocon-associated protein subunit beta), M8A2R3 (Uncharacterized protein/Ricin B-like lectin EULS3-like family), M7Z7R6 (Uncharacterized protein/isoflavone reductase), and Q6RUJ1 (Glutamine synthetase), presented a pattern of accumulation, with a continuous increase over development. Conversely, Q6RUJ1 (Glutamine synthetase) had an opposite accumulation pattern (decreasing from 300 to 600°C days after anthesis). The accumulation of M7Z7R6 (Uncharacterized protein/isoflavone reductase) was constant over time. Considering the response of the AGs to the different N/S supplies, we could observe that these four proteins had different profiles. M7Y8M9 (Translocon-associated protein subunit beta), M8A2R3 (Uncharacterized protein/Ricin B-like lectin EULS3-like family), contents decreased with S supply whereas Q6RUJ1 (Glutamine synthetase) and M7Z7R6 (Uncharacterized protein/isoflavone reductase) ones increased under the two high S treatments (S and NS). However, the accumulation for the latter AG is significantly increased by high N supply (treatment N).

### Association Study for Statistical Validation of the 18 AGs Highlighted by DIABLO Approach

To validate the results evidenced in our data analysis, a genetic association study was done by searching in the bread wheat genome for the orthologous and paralogous genes coding for the 18 AGs highlighted in the network. A search for genes coding the 18 AGs included in the network was carried out in bread wheat genome (Supplementary Table 1). Eighty-eight loci orthologous or paralogous were detected. All chromosomal groups were represented except the group 6. Using coordinates of each gene, we extracted 181 SNP markers and 11 OTV from the BreedWheat set (Supplementary Table 1). After filtering, 126 markers in 34 genes out of 81 (42%) have been used for analysis. No marker was available in the gene coding for M8A2R3, M7ZQM3 and M7ZAQ9.

Significant associations (*P* < 0.001) were found in a least one of the homologous gene coding for five AGs (D0PRB4, I0JTT9, M7YBY6, M7Z2B3, and M7Z7R6) (Table 2). D0PRB4 (Peroxiredoxin) was associated with the quantity of ω-gliadin per grain; I0JTT9 (Glutamate decarboxylase) to the quantity of γ-gliadin per grain; M7YBY6 (Basic endochitinase C) to quantities per grain of LMW glutenins and gliadins (especially the α-gliadin); M7Z2B3 (Uncharacterized protein/transmembrane 214-B) to the quantity of HMW glutenins per grain and thus to the HMW to LMW glutenin ratio; M7Z7R6 (Uncharacterized protein/isoflavone reductase) to the quantity of ω-gliadin per grain. Therefore, linkage mapping confirmed the role of some AGs in the synthesis and accumulation of SPs. It is worth noting that associations found for M7Z2B3 (Uncharacterized protein/transmembrane 214-B) perfectly fitted the network produced by DIABLO analysis (Figure 5).

**Table 2:** Association analysis. Proteins from the network associated to storage protein composition.

Uniprot ID	Chr	HMWtoLMW	HMW	LMW	Gliadin	α_gliadin	γ_gliadin	ω_gliadin
D0PRB4	2B							X
I0JTT9	3A						x	
M7YBY6	7A			x				
M7YBY6	7B				x	x		
M7Z2B3	3A	x	x					
M7Z7R6	1A							X


*Uniprot ID, accession Uniprot; Chr, chromosome; HMWtoLMW, HMW glutenins to LMW glutenins ratio; HMW, LMW, Gliadin, quantity of storage proteins by grain (mg N grain^-*1*^); α_, γ_, ω_ gliadin, percentage of alpha-, gamma-, omega-gliadin in total gliadins.*

## Discussion

From a dataset of 352 AGs, we were able to take into account the effect of the combination of stage of development and N and S supply to identify the most discriminating proteins associated with SP composition using sparse methods. Five of these proteins were statistically validated by genetic association analysis. These results demonstrate the capacity of these new methods to identify candidates for further investigations, especially functional validation. Due to the clear structuration of the data that was found to be related to developmental stage and nutrition, quantitative data were driven using these two factors. Following the identification of the most discriminating AGs, a network was established to focus on the candidate AGs highly correlated to SPs.

### Input of the Integrative Approach

**FIGURE 6:**
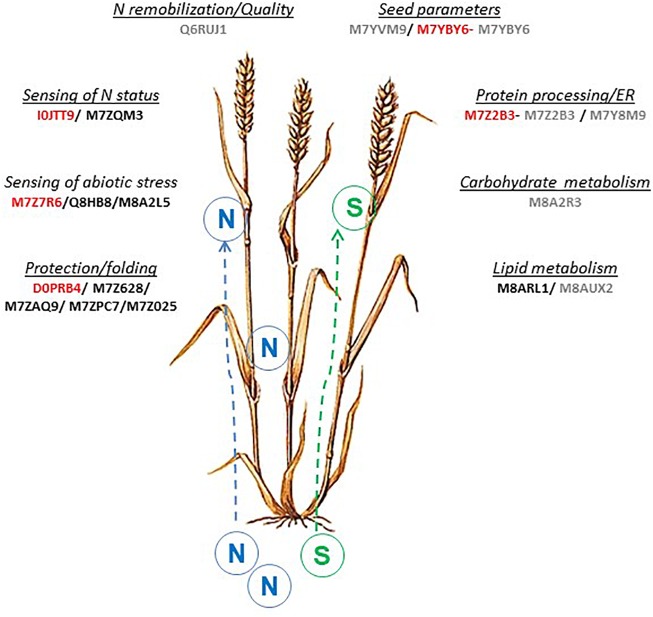
Overview of AGs proteins and related pathways involved in wheat grain development associated with N or S supply. The 18 AGs proteins highlighted by the integrative approach were indicated, in red: 5 AGS proteins also found by association analysis (this paper) and in gray: 7 AGs proteins found by rules analysis network approach (Bonnot et al., 2017).

Data-mining methods are diverse. For this study, we used a multivariate projection methodology developed in DIABLO to produce a network, which includes 18 AGs and 7 variables related to seed SPs composition. Roughly, these 18 AGs are involved in N metabolism, stress sensing, storage metabolism and, protein processing or folding (Figure 6). Other methods for data integration are available such as methods based on the discovery of association rules between attributes (Agier et al., 2007; Vincent et al., 2015). This method has been used to find associations between proteins and to compare their behavior between treatments by Bonnot et al. (2017). The network obtained by these authors included several AGs, including 7 AGs (M7YBY6, M7YVM9, M7Z2B3, M8AUX2, M8A2R3, M7Y8M9, and Q6RUJ1) that were also highlighted by the multivariate projection methodology (Figure 6). The statistical validation carried out using genetic association analysis confirmed the putative role of 5 candidate AGs out of the 18, D0PRB4, I0JTT9, M7YBY6, M7Z2B3, M7Z7R6, in the synthesis of SPs (Figure 6). M7YBY6, annotated as an endochitinase, and M7Z2B3 (involved in the reticulum stress apoptosis) are identified by both integrative methods and significantly associated with SPs. Altogether, the integration of AG and SP datasets provided firm and consistent results on the relationship between some AGs and SPs. A few central components have been highlighted. Therefore, biological interpretation of the network could be helpful to improve understanding of the SP synthesis.

### Biological Interpretation

Nutrients, stress, hormones, and growth factors all affect protein biosynthesis through complex signaling pathways. Although the synthesis of AGs is less affected by N than that of SPs (Wieser and Seilmeier, 1998; Martre et al., 2003; Plessis et al., 2013), N and S nutrition has been reported to affect several enzymes in wheat grain (Flæte et al., 2005). Recently, Bonnot et al. (2017) have shown that the grain proteome was impacted by the N/S balance. Our results revealed dynamic enzyme activities related to the grain development (four stages during the early phases of endosperm cell division and cell differentiation) and the N/S supply (four nutrition supplies). The early phases of grain development determine the potential final size of the grain (Xie et al., 2015) and are particularly sensitive to environmental conditions. The multivariate projection method used in this work revealed variables from each dataset (AGs and SPs) mainly involved in discrimination according to the combination of N and S supply and grain developmental stage (Figure 4B). Eighteen AGs and seven SP variables important in discrimination were highly correlated as shown by the network. Based on the principle of “guilt per association,” these AGs could influence the synthesis of SPs in relation to developmental stage and nutrition as confirmed for a few of them by linkage mapping. These proteins are involved in sensing processes, N assimilation, protein protection or folding, and storage metabolism.

### Sensing of Abiotic Stress

Therefore, several AGs linked to SPs have a function related to S or N starvations [M7Z7R6 (Uncharacterized protein/isoflavone reductase), Q6RUJ1 (Glutamine synthetase), I0JTT9 (Glutamate decarboxylase), and M7ZQM3 (Globulin-1 S allele)].

M7Z7R6 has similarities with an isoflavone reductase. In maize (*Zea mays*), a gene highly homologous to isoflavone reductase has been identified; it encodes an NADPH binding enzyme and is activated in response to S starvation (Petrucco et al., 1996). The glutathione-dependent regulation indicated that maize isoflavone reductase like genes may play a crucial role in the establishment of a thiol-independent response to oxidative stress under glutathione shortage conditions. The identification of genes activated in S starvation may thus provide insights not only into the mechanisms of adaptation to nutrient limitation but also into the response to stress resulting from glutathione depletion. In rice, an isoflavone reductase-like gene has been shown to be involved in homoeostasis of reactive oxygen species (Kim et al., 2010). In wheat, detailed physiological dissection of this gene is underway to identify the underlying mechanism conferring heat tolerance (Singh et al., 2018). The location of M7Z7R6 in the correlation circle (Figure 4B) at the right of the *x*-axis confirmed the role of this metabolic protein in response to S deficiency. This AG is over-expressed when S is lacking whatever is the developmental stage of the grain (Supplementary Figure 4). As expected, M7Z7R6 is negatively correlated with S rich SPs, α-, γ- and total-gliadins (Figure 4, 5).

Glutamine synthetase (Q6RUJ1) plays a major role in the assimilation of ammonia during remobilization of N to the grain (Bernard et al., 2008; Zheng et al., 2018). Q6RUJ1 projection (Figure 4) indicates that this enzyme is more expressed in the early development stages and in nutrition conditions with high S (NS and S). The abundance of this protein was highly correlated with that of HMW-GS.

Glutamate decarboxylase is a key enzyme of the GABA pathway (Miflin and Habash, 2002). GABA has been associated with various physiological responses (Carillo, 2018), including N starvation (Recht et al., 2014) and sensing of N status (Lancien and Roberts, 2006). In plants, GABA increase is a common response to a restriction of glutamine synthesis, reduction of protein synthesis and an increase in protein degradation (Bouché and Fromm, 2004), which happen concomitantly. GABA is involved in 14-3-3-genes regulation (Lancien and Roberts, 2006). Shin et al. (2011) have reported the role of 14-3-3 proteins in coordinating carbon (C), S, and N metabolism through the regulation of several key enzymes including glutamine synthetase (Q6RUJ1) and glutamate decarboxylase (IOJTT9).

Globulin 1S allele (M7ZQM3) was correlated to gliadin to glutenin ratio. The gene coding for this protein was identified by Zheng et al. (2018) in response to N application.

In addition, we detected two other proteins related to abiotic stresses (M7YBY6 and M7YVM9). This is not surprising as many proteins with stress and defense-related ontologies have been reported to be upregulated during grain development (Nadaud et al., 2010; Capron et al., 2012; Ma et al., 2014; Brinton and Uauy, 2018).

### Adaptative Response of the Grain to N and S Supply

During grain filling, two major processes, storage synthesis and accumulation take place. They concern starch and SPs. Grain SPs were synthesized on the rough endoplasmic reticulum (ER) and then passed into the lumen, where cleavage of signal peptides, formation of intra- and inter-chain disulphide bonds, folding, assembly and aggregation of polypeptides happen (She et al., 2011). These processes are assisted by molecular chaperons (Bertolotti et al., 2000) and chaperon proteins in the ER lumen are crucial for the biogenesis of SP bodies (She et al., 2011).

During the processing, chaperone proteins like M7ZPC7 (Heat shock cognate 70 kDa protein) transiently bind many nascent secretory proteins and probably prevents non-specific aggregation to facilitate their folding and assembly (Muench et al., 1997). Saito et al. (2009) showed that binding proteins were involved in rice prolamin deposition in ER despite the absence of the retention signal.

Peroxiredoxins (D0PRB4 and M7Z628) are thiol peroxidases with multiple functions in the antioxidant defense and redox signaling network of the cell. They could also act as chaperons (Chae et al., 2012; Lee S.L. et al., 2018).

In eukaryotic cells, one-third of all proteins must be transported across or inserted into the endoplasmic reticulum (ER) membrane by the ER protein translocon. The translocon-associated protein (TRAP) complex is an integral component of the translocon (Pfeffer et al., 2016). The protein M7Y8M9, a subunit of the protein complex located in the ER membrane, could act in this process.

Carbohydrate and lipid metabolism could be modified by both development and nutrition status as previously described (Cao et al., 2016; Bonnot et al., 2017; Yang et al., 2017). M8ARL1 and M8AUX2 (caleosin) were related to lipid metabolism. In *Arabidopsis*, seed-specific caleosins are viewed as oil-body associated proteins that possess Ca (2+)-dependent peroxygenase activity and are involved in processes of lipid degradation (Poxleitner et al., 2006). In *Arabidopsis* some seed-specific caleosins had peroxygenase activity (Partridge and Murphy, 2009).

Lee K.H. et al. (2018) showed that grain width 2 (GW2) strongly interacted with chitinase 14 (CHT14). GW2 homolog has been identified in wheat (*T. aestivum*; TaGW2). Like in rice, this protein is involved in the regulation of grain weight and width at maturity (Su et al., 2011). M7YBY6 and M7YVM9, which have a chitinase activity, could influence these parameters and indirectly the storage capacity of the grain.

### The Link With the End-Used Quality of the Flour

If AGs are rich in S-containing amino acids (Zhao et al., 1999) and could influence directly the rheological properties of wheat flour (Hill et al., 2008), they also have an indirect role by influencing the SP content and composition, which are the main determinants of flour end-used (Zheng et al., 2018). As seen above, all the AGs included in the network obtained in this work could be biologically related to SP synthesis. They are likely indirect components of wheat end-used quality.

This is probably the case for Q6RUJ1 (glutamine synthetase) as reported by Gao et al. (2009), who showed that the abundance of this enzyme is higher at the early period of grain development in a good bread making quality cultivar than in a poor one. This AG could influence the flour quality by two ways. First, a high expression level of this enzyme corresponds to a high quantity per grain of HMW glutenins as indicated by the strong positive correlation between these two variables. HMW glutenins constitute the backbone of the gluten network. Consequently, they are a major determinant of the rheological properties of dough and influence its mixing properties (Shewry et al., 2003). Secondly, the high expression of glutamine synthetase at early developmental stage of the grain was reported to facilitate the folding of gluten proteins producing more regular glutenin polymers, which might improve the gluten quality (Miflin and Habash, 2002).

The gliadin to glutenin ratio is related to a measure of molecular weight distribution. At constant protein content, decreases in the gliadin to glutenin ratio were associated with several dough parameters with, for examples, increases in dough mixing time, maximum resistance to extension, and loaf volume and with decreases in extensibility (Uthayakumaran et al., 1999). Therefore, its optimal value depends on the process: low values are required for bread making while high values are advantageous for pastries (Marchetti et al., 2012; Barak et al., 2014). Here, we found many AGs able to help manage this ratio depending on the nutrition and developmental stage. It could be increased by a higher expression of M8AUX2 (Uncharacterized protein/caleosin 2), which concomitantly led to a decrease of HMW glutenins.

## Conclusion

In this study, we used new tools based on multivariate statistical approaches to unravel the relationship between AGs and SPs known to influence the end-used quality of wheat flour. The results highlighted the potential role of 18 AGs in SP synthesis, which are consistent with their annotation. In addition, 12 AGs (67%) of this set have been confirmed as important components for SP synthesis by an integrative methodology based on rules or linkage mapping. These results demonstrated the efficiency of the approach used especially since the statistical validation had been limited by the lack of markers in some sequences. Therefore, the number of AGs statistically validated might be underestimated. Two AGs (M7YBY6, an endochitinase, and M7Z2B3, involved in the reticulum stress apoptosis) were identified by all the strategies. The latter is overexpressed in S-deficient conditions. This enzyme contributes to increase the gliadin to glutenin ratio by decreasing the HMW glutenin quantity per grain. The down-accumulation of this enzyme might increase HMW glutenin quantity and then the baking quality when the level of fertilization is low. However, the role of this AG must be functionally validated.

Thus, we have shown the interest of multivariate statistical approaches to extract the most significant variables from large datasets in response to abiotic constraints. Interestingly, these methods are able to consider the whole dataset instead of using only differentially expressed variables because the hypotheses rest on similar expression patterns across a set of samples indicating functional relationships. This has an important biological meaning because variables (AGs), which are not differentially expressed during grain development or in response to N or S supply, may be important actors due to interactions with other proteins (which may be differentially expressed). These results also highlight the consistency and complementarity of the approaches and the need to take into account the proteins obtained by each of them to get a comprehensive view. To know if changes observed could be found also at a transcriptional level, transcriptomic approaches would be necessary. However, as RNA and protein abundances are weakly correlated in wheat (about 32%, Tasleem-Tahir, 2012), we would not necessarily obtained the same response.

To conclude, the innovative method used here was shown to provide an operational framework to biologists, who can then follow only a few candidate entities for functional validation. Therefore, further work has to be initiated to see how the targeted AGs influence the SP synthesis and the quality parameters in relation to N and S fertilizer applications. Their implementation in future breeding programs has also to be investigated.

## Data Availability

The datasets generated for this study can be found in the ProteomeXchange Consortium, PXD006058.

## Author Contributions

PM, EB, and CR conceived and coordinated the study. EB and CR carried out the statistical analysis. SD helped for the modeling and the optimization methods. DA collected the phenotypic data. MD, TB, EB, and MZ carried out the proteomic analysis. EB, TB, and CR wrote the manuscript. All authors edited and approved the final version of the manuscript.

## Conflict of Interest Statement

The authors declare that the research was conducted in the absence of any commercial or financial relationships that could be construed as a potential conflict of interest.

## Footnotes

1 www.thegpm.org/tandem

2 https://www.wheatgenome.org/
