# Supplementary figures and images for: Proteomic Data Integration Highlights Central Actors Involved in Einkorn (Triticum monococcum ssp. monococcum) Grain Filling in Relation to Grain Storage Protein Composition
Source: Front Plant Sci. 2019 Jul 4;10:832. doi: 10.3389/fpls.2019.00832 (PMC6620720; doi:10.3389/fpls.2019.00832)

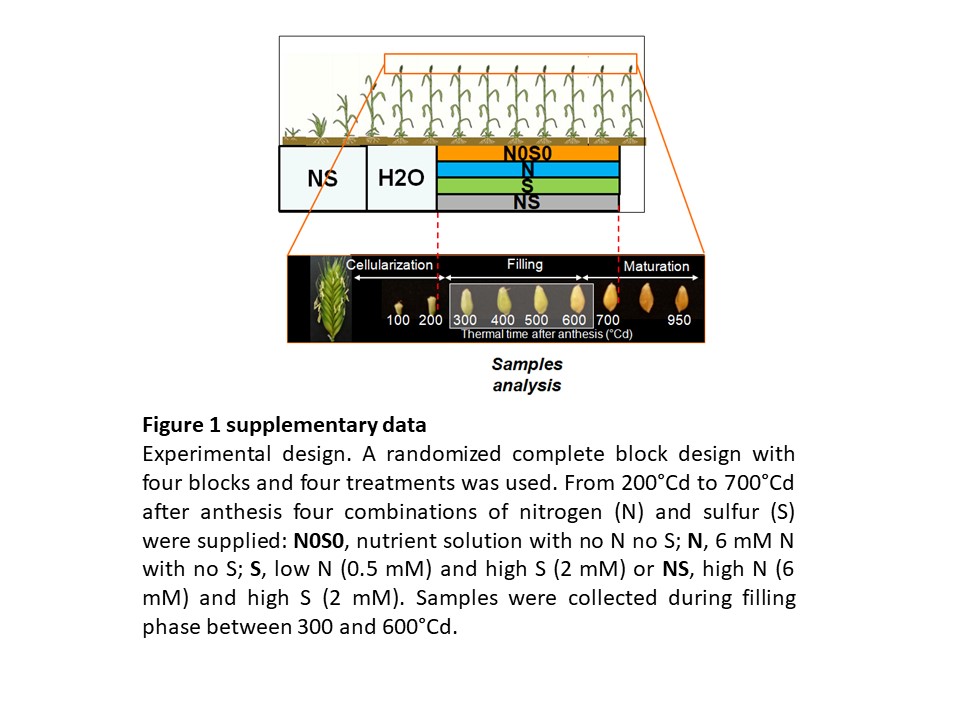

Supplement: Supplementary file 2 [file Image_1.jpg]

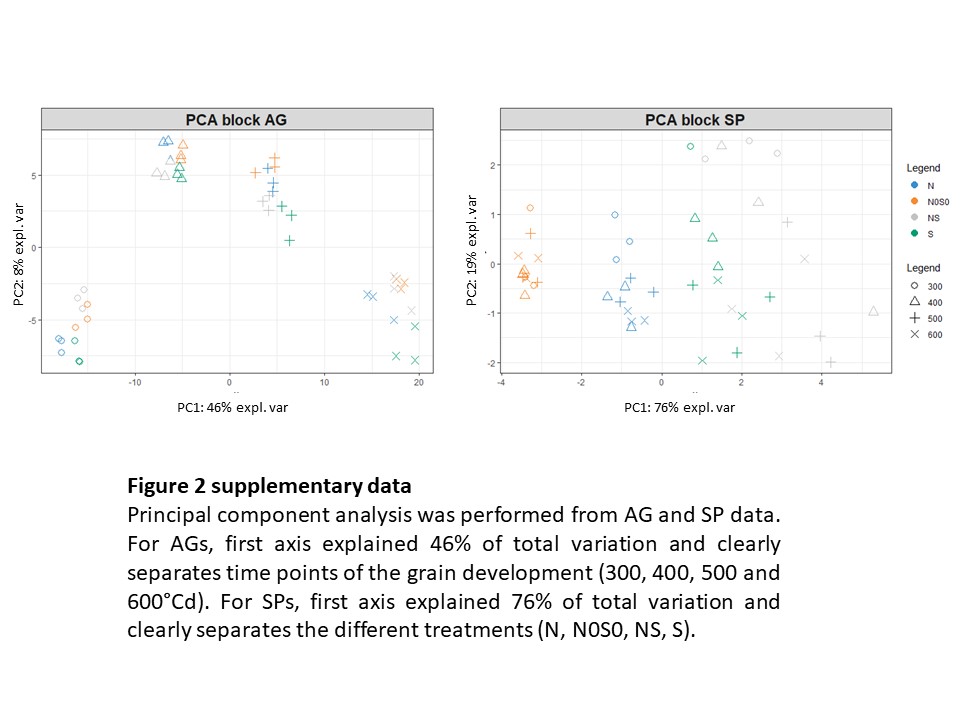

Supplement: Supplementary file 3 [file Image_2.jpg]

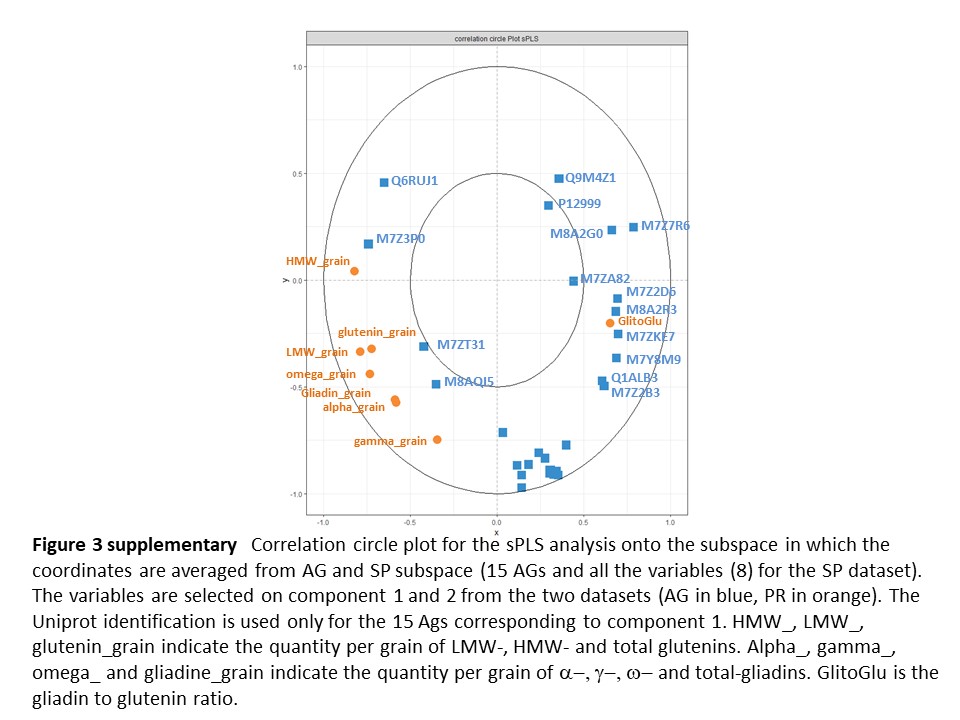

Supplement: Supplementary file 4 [file Image_3.jpg]
