# Supplementary material for: Proteomic Data Integration Highlights Central Actors Involved in Einkorn (Triticum monococcum ssp. monococcum) Grain Filling in Relation to Grain Storage Protein Composition
Source: Front Plant Sci. 2019 Jul 4;10:832. doi: 10.3389/fpls.2019.00832 (PMC6620720; doi:10.3389/fpls.2019.00832)
Supplement: Supplementary file 5 [file Image_4.pdf]

A

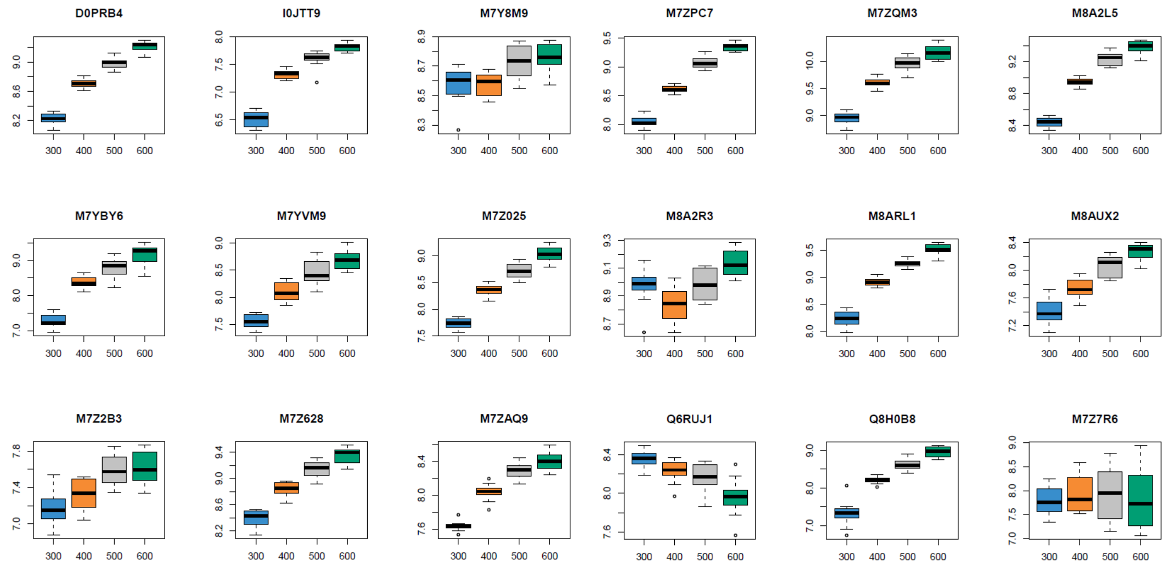

B

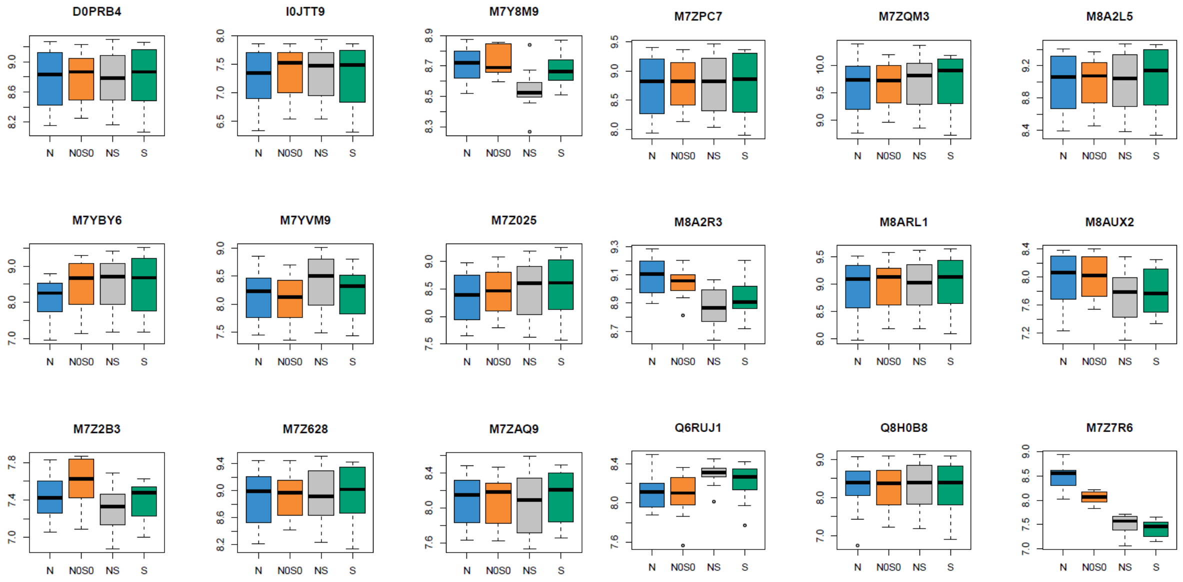

Supplementary Figure 4 Effects of stage (A) or N and S (B) supply on the quantity of AGs present in the network.

Development stages are expressed as thermal time after anthesis : 300 (blue), 400(orange), 500 (grey) or 600°C (green) days.

Nutrition status are no N and no S (N0S0, orange); N with no S (N, blue); S with low N (S, green) or high N and high S (NS, grey)
